# Investigation of Bacteriocin Production Ability of *Pediococcus acidilactici* JM01 and Probiotic Properties Isolated From Tarak, a Conventional Korean Fermented Milk

**DOI:** 10.1002/fsn3.71003

**Published:** 2025-09-22

**Authors:** Hyunwoo Ahn, Hyun Jun Lee

**Affiliations:** ^1^ Department of Food Science and Biotechnology Dongguk University‐Seoul Goyang‐si Republic of Korea; ^2^ Department of Food Engineering Mokpo National University Republic of Korea

**Keywords:** bacteriocin, *Pediococcus acidilactici* JM01, probiotics, tarak

## Abstract

This study aimed to isolate and characterize a bacteriocin‐producing lactic acid bacterium from Tarak, a traditional Korean fermented milk, for potential applications in food safety and health. *Pediococcus acidilactici* JM01 was identified and shown to produce a bacteriocin with strong antimicrobial activity against *Listeria monocytogenes* . The maximum activity (2560 AU/mL) was observed after 12 h of incubation and remained stable across a broad pH range (2–11) and under various heat treatments. The bacteriocin was inactivated by proteolytic enzymes and had an estimated molecular weight of 6 kDa. A plasmid‐curing test confirmed plasmid involvement in bacteriocin production. Furthermore, JM01 exhibited resistance to acidic and bile conditions, strong adhesion to epithelial cells, and anti‐inflammatory effects by suppressing the expression of IL‐1β, IL‐6, TNF‐α, and MCP‐1. Collectively, these findings demonstrate that *P. acidilactici* JM01 possesses both antimicrobial and probiotic properties, highlighting its potential as a functional probiotic for enhancing food safety and promoting health.

## Graphical abstract

This study aimed to isolate and characterize a bacteriocin‐producing lactic acid bacterium, *Pediococcus acidilactici* JM01, with probiotic potential from Tarak.

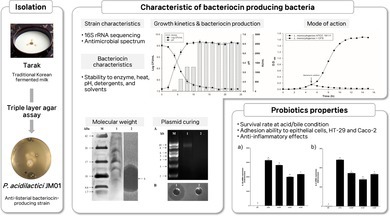

## 1 Introduction

*Tarak*, a traditional Korean fermented milk, holds significant historical and cultural value, as documented in the ancient Korean cookbook ‘Soowoonjapbang’ written around AD 1500. This fermented milk is produced by adding makgeolli, a Korean rice wine, to boiled milk, followed by fermentation at a warm temperature of up to 37°C (Osada et al. 2014). Recent research has highlighted a rich, diverse range of microorganisms in Tarak, including *Lactobacillus, Leuconostoc, Saccharomyces*, and *Pichia* genera (Lim et al. 2013).

A wide range of traditional fermented foods, including yogurt, kefir, kimchi, sauerkraut, and fermented sausages, is recognized as rich reservoirs of lactic acid bacteria (LAB). These microorganisms contribute not only to the sensory attributes and shelf life of fermented products but are also associated with health‐promoting effects through their probiotic properties and production of antimicrobial metabolites, including organic acids, hydrogen peroxide, and bacteriocins (Castellone et al. 2021; Marco et al. 2017; Rhee et al. 2011). Among these, dairy‐based fermented foods have received considerable attention as sources of bacteriocin‐producing LAB, which hold significant potential for applications in food bio‐preservation and gut health.

*Listeria monocytogenes* , a psychrotrophic Gram‐positive pathogen, is capable of surviving under refrigeration, high salt, and low pH conditions, and can persist in food processing environments through biofilm formation (Osek and Wieczorek 2023). It causes listeriosis, a serious foodborne illness with a high fatality rate, particularly affecting pregnant women, neonates, the elderly, and immunocompromised individuals, and is therefore a major concern for food safety in both ready‐to‐eat and fermented foods. Although lactic acid fermentation generally improves microbial safety, *Lis*. *monocytogenes* has been shown to proliferate in chicken‐based dry‐fermented sausages when a bioprotective LAB starter culture is not applied, whereas its growth is effectively suppressed when such protective cultures are used (Austrich‐Comas et al. 2022). Therefore, controlling specific pathogens such as *Lis*. *monocytogenes* through natural antimicrobial strategies, including protective LAB, is essential to ensure the microbial safety of fermented foods. In this context, isolating bacteriocin‐producing LAB from traditional fermented foods like Tarak and characterizing their functional properties is a promising approach to address both food safety and health‐related challenges.

Ribosomally synthesized antimicrobial peptides, known as bacteriocins and produced by LAB, have attracted considerable interest for their potential in food bio‐preservation and as natural alternatives to conventional antibiotics (Bisht, Das, Hussain, et al. 2024; Bisht, Das, and Navani 2024). These antimicrobial peptides, effective against a wide range of Gram‐positive/negative foodborne pathogens and spoilage organisms, are generally recognized as safe (GRAS). Research into the classification, antibacterial mechanisms, and food safety applications of bacteriocins is a promising field, with novel screening methods and technological advances aiding in the discovery of new bacteriocins (Lahiri et al. 2022).

Bacteriocins can be leveraged to modulate diseases induced by pathogenic, including multi‐drug‐resistant, bacteria (Huang et al. 2021). The use of bacteriocins in humans is considered safe because they are degraded by proteolytic enzymes in the stomach (Ahn, Lee, Lee, Kim, and Lee 2023). Consequently, the colonization of bacteriocin‐producing probiotics in the GI tract could prevent pathogenic bacterial invasion through bacteriocin production and modulation of the host's immune responses (Bu et al. 2022). These attributes underscore the importance of isolating bacteriocinogenic LAB from *Tarak* and conducting a preliminary evaluation of both their probiotic potentials and antimicrobial potentials.

## 2 Materials and Methods

### 2.1 Isolation and Identification of Bacteriocin‐Producing Strain

Bacteriocin‐producing lactic acid bacteria were isolated from traditional homemade *Tarak*, kindly provided by Do Eun Kim, the chief daughter‐in‐law of the Seolweoldang Jongga, the leading family of Andong. The ‘Triple agar layer’ technique was utilized to isolate these bacteria as reported in previous studies (Ahn, Lee, Lee, and Lee 2023), using *Lis. monocytogenes* ATCC 15313 as the indicator strain.

The isolated strain was identified using cell morphology, Gram staining, catalase test, API 50 CHL kit (Biomérieux, Marcy l'Etoile, France), and 16 s rRNA sequencing. The 16 s rRNA sequences were amplified with universal primers 27F and 1492R, and the amplified PCR products were confirmed through Sanger sequencing conducted at Macrogen Inc., Seoul, Republic of Korea. Related bacterial sequences to the isolate's sequence were searched in the GenBank database using the BLAST tool, and a phylogenetic tree was constructed by the neighbor‐joining method with 2000 bootstrap replicates using MEGA 11.0 software.

### 2.2 Growth Kinetics and Bacteriocin Production

The isolate was cultured in MRS broth at 37°C for 24 h with 1% inoculum. Viable cell counts (CFU/mL) and pH were monitored every 2 h. Additionally, bacteriocin production over time was assessed against *Listeria monocytogenes* ATCC 15313 using the agar‐well diffusion method. Bacteriocin activity was measured in arbitrary units (AU) per mL, defined as the reciprocal of the highest dilution showing the inhibition zone.

### 2.3 Effect of Enzymes, Temperature, pH, Detergents and Solvents on Bacteriocin

The isolate was cultivated in MRS broth at 37°C for 20 h. The CFS was prepared as described above, neutralized to pH 6.5 with 5 N‐NaOH or HCl, and then membrane‐filtered. Enzymes such as trypsin, pronase E, α‐chymotrypsin, α‐amylase, lipase, catalase (Sigma‐Aldrich, St. Louis, MO, USA), and RNase A, proteinase K (Intron Biotech Inc., Korea) were prepared in 50 mM sodium phosphate buffer (pH 7.0), mixed with CFS at a final concentration of 0.1 mg/mL, and incubated at 37°C for 2 h.

Thermal stability of bacteriocin was tested by heating at 60°C, 80°C, and 100°C for 30 min, and by autoclaving at 121°C for 15 min. Samples were cooled to room temperature prior to the antimicrobial activity assay. To assess the effect of pH on bacteriocin, the CFS was adjusted to varying pH levels (2, 3, 5, 7, 9, 11, and 12) and then incubated at 37°C for 2 h.

The effect of detergents and solvents on bacteriocin stability was evaluated by adding various detergents, such as Sodium dodecyl sulphate (SDS, 1%, 2%, w/v), Tween 20 (1%, w/v), and Tween 80 (1%, w/v), along with solvents like methanol, ethanol, iso‐propanol, acetone, and chloroform (50%, v/v). Following all treatments, samples were readjusted to pH 6.5 and membrane filtered for assay. The antimicrobial assay of treated samples was conducted using the agar well diffusion method, with *Listeria monocytogenes* ATCC 15313 serving as the indicator.

### 2.4 Molecular Weight Determination of Bacteriocin JM01

The molecular weight of the partially purified bacteriocin produced by the isolate was determined by using partially purified of the cell‐free supernatant (CFS). Initially, the isolate was inoculated (1%, v/v) into MRS broth and incubated for 20 h at 37°C. The CFS was collected through centrifugation (19,461 × g, 15 min, 4°C) followed by membrane filtration. Proteinaceous compounds in the CFS were precipitated using 70% ammonium sulfate. Subsequently, the precipitate was gathered by centrifugation (Ahn, Lee, Lee, and Lee 2023). The precipitate was resuspended in deionized water and dialyzed using benzoylated dialysis tubing (molecular weight cut off 2000, Sigma‐Aldrich, St. Louis, MO, USA). The molecular weight of the partially purified bacteriocin was assessed using tricin SDS‐PAGE with a 16% separating gel (Ortiz‐Rodríguez et al. 2023). Post‐electrophoresis, to determine the bacteriocin's molecular weight, one gel was stained with a silver staining kit (Thermo Fisher Scientific, Waltham, MA, USA), while the other gel was transferred aseptically into a petri dish and overlaid with *Lis*. *monocytogenes* ATCC 15313 seeded agar (10^6^ CFU/mL) to measure the inhibition zone.

### 2.5 Plasmid Curing

Novobiocin (Sigma‐Aldrich, St. Louis, MO, USA) was utilized as a curing agent to eliminate indigenous plasmids from the isolate. The highest sub‐lethal concentration of novobiocin allowing growth at 37°C was determined to be 1.0 μg/mL. To obtain a plasmid‐cured strain, the isolate was inoculated (10^3^ CFU/mL) in MRS broth with a sub‐lethal dose of novobiocin (nMRS). The culture was sub‐cultured three times in nMRS broth at 37°C, at 48 h intervals. Bacteriocin‐negative (Bac^−^) colonies were identified using the ‘Triple agar layer’ method described above. Plasmids were extracted using the plasmid extraction kit (Fast DNA‐spin Plasmid DNA Purification Kit, Intron Biotech Inc., Korea) and compared with those from a bacteriocin‐producing (Bac^+^) strain via agarose gel electrophoresis. A supercoiled 1 kb DNA ladder (Elpisbiotech, Korea) was used as a marker. The linkage of bacteriocin production to plasmids was assessed through an agar well diffusion assay using pH‐adjusted CFS from both Bac^+^ and Bac^−^ strains.

### 2.6 Antimicrobial Spectrum

The antimicrobial spectrum of the isolate was performed by a deferred assay and an agar‐well diffusion assay as detailed in a previous publication (Ahn et al. 2017). Table 1 presents the 37 test microorganisms (28 Gram‐positive bacteria, 7 Gram‐negative bacteria, and 2 yeast), including the media and incubation temperatures used. Following incubation, antimicrobial activity was assessed by measuring the diameter of the inhibition zones. The antimicrobial activity against the indicator strain was expressed as inhibition zone diameter: + for 1–10 mm, ++ for 11–20 mm, and +++ for over 21 mm.

**TABLE 1:** Antimicrobial spectrum of *P. acidilactici* JM01.

Microorganism	Media	Temp (°C)	DA	AW
Gram positive				
*Bacillus cereus* KCTC 1012	NA	30	−	−
*Bacillus mycoides* KCTC 3453	NA	25	−	−
*Bacillus subtillis* KCTC 2023	NA	30	+	−
*Bacillus thuringiensis* KCTC 3452	NA	30	−	−
*Lactobacillus acidophilus* KCTC 3179	MRS	37	+	−
*Levilactobacillus brevis* KCTC 3498	MRS	30	+	+
*Lacticaseibacillus casei* KFRI 704	MRS	37	+	−
*Latilactobacillus curvatus* KCCM 43119	MRS	30	+	+
*Latilactobacillus curvatus* KCTC 3767	MRS	30	++	+
*Lactobacillus delbrueckii subsp. delbrueckii KFRI 154*	MRS	30	+	−
*Lactobacillus johnsonii* ATCC 11506	MRS	37	+	−
*Lactiplantibacillus plantarum* NCDO 955	MRS	37	+	−
*Lactiplantibacillus plantarum* KFRI 814	MRS	37	+	−
*Latilactobacillus sakei* subsp. *sakei* KCTC 3603	MRS	30	+++	+++
*Leuconostoc inhae* KCTC 3774	MRS	25	++	++
*Leuconostoc mesenteroides* ATCC 10830	MRS	30	−	−
*Leuconostoc mesenteroides* KCCM 43060	MRS	30	++	+
*Leuconostoc pseudomesenteroides* KCTC 3652	MRS	30	+	+
*Listeria innocua* ATCC 33090	BHI	37	++	−
*Listeria monocytogenes* KCTC 13064	BHI	37	+++	+++
*Listeria monocytogenes* ATCC 19111	BHI	37	+++	+++
*Pediococcus pentosaceus* IFO 3884	MRS	37	++	+
*Propionibacterium acnes* ATCC 6919	RCM	37	+	−
*Staphylococcus aureus* KCTC 3881	NA	37	+	−
*Staphylococcus epidermidis* KCTC 3958	NA	37	+	−
*Streptococcus mutans* ATCC 25175	BHI	37	+	−
*Weissella cibaria* KCTC 3746	MRS	30	+	−
*Weissella koreensis* KCCM 41517	MRS	30	+	−
Gram negative				
*Escherichia coli* O157:H7 ATCC 35150	EC	37	++	−
*Cronobacter sakazakii* KCTC 2949	NA	37	+	−
*Klebsiella pneumoniae* KCCM 40890	NA	37	+	−
*Pseudomonas aeruginosa* KCTC 2004	NA	37	+	−
*Salmonella enterica* subsp. *enterica* ser. Enteritidis KCCM 12021	NA	37	+	−
*Salmonella enterica* subsp. *enterica* ser. Typhimurium KCTC 11806	NA	37	+	−
*Shigella sonneii* KCCM 40947	NA	37	+	−
Yeast				
*Candida albicans* KCTC 7270	YPD	25	−	−
*Saccharomyces cerevisiae* ATCC 13264	YPD	25	−	−

*Note:* Diameter of inhibition zone; −, absence of inhibition zone; +, 1–10 mm; ++, 11–20 mm; +++, > 21 mm.

Abbreviations: AW, agar well diffusion assay; DA, deferred assay.

### 2.7 Mode of Action

*Lis*. *monocytogenes* ATCC 15313 was inoculated (1% inoculum) in 80 mL of BHI broth and incubated at 37°C. When the growth of *Lis*. *monocytogenes* ATCC 15313 reached an early exponential phase (at 4 h), 20 mL of the isolate's CFS (adjusted to pH 6.5, 2560 AU/mL) was added. Optical density was read hourly at 600 nm using UVmini‐1240 (Shimadzu, Kyoto, Japan).

### 2.8 Acid and Bile Tolerance

The acid and bile tolerance of the isolate was evaluated. Briefly, for acid tolerance, the isolate was cultured overnight, centrifuged, and washed once with phosphate‐buffered saline (PBS) before being resuspended at a concentration of 10^8^ CFU/mL in 10 mL of MRS broth adjusted to pH levels between 1.5 and 4.0. The cells were incubated at 37°C for 3 h, during which time viable cell counts were enumerated every 90 min using MRS agar. For bile tolerance, cells were resuspended in 10 mL of MRS broth containing porcine bile extract concentrations ranging from 0.3% to 2.0% (w/v) (Sigma‐Aldrich, St. Louis, MO, USA) and incubated at 37°C for 4 h; viable cell counts were monitored using the plate count method on MRS agar. Survival rates were calculated by comparing initial and final viable cell counts.

### 2.9 Adhesion Ability to Human Intestinal Epithelial Cell Line HT‐29 and Caco‐2

The adhesion ability of the isolate to human epithelial cell lines HT‐29 and Caco‐2, obtained from the American Type Culture Collection (ATCC), was assessed. Both cell lines were cultivated in Dulbecco's modified Eagle's medium (DMEM, Welgene, Gyeongsangbuk‐do, Korea) supplemented with 10% fetal bovine serum (FBS, Gibco, Burlington, ON, Canada), 100 μg/mL streptomycin, and 100 U/mL penicillin (Gibco) at 37°C in 5% CO_2_.

Both cells were plated in 24‐well culture plates and grown to full confluence. Cells were washed with PBS to remove residual antibiotics. *P. acidilactici* JM01 was centrifuged, washed three times with PBS, and then re‐suspended in antibiotic‐free DMEM. *P. acidilactici* JM01 was inoculated (10^9^ CFU/mL) into both cell lines. After incubating at 37°C for 1 h, the plates were washed three times with PBS. 0.2% Triton‐X100 (Sigma‐Aldrich, St. Louis, MO, USA) was used to detach adherent bacteria from HT‐29 and Caco‐2 cells. The number of adherent bacterial cells was measured by the plate count method in MRS agar.

### 2.10 Anti‐Inflammatory Effects of *P. acidilactici* JM01 on LPS‐Induced RAW 264.7

RAW 264.7 cells were seeded in a 6‐well plate with 5 × 10^5^ cells/ml. When the cells reached 70% confluence, LPS (at a final concentration of 1 μg/mL) and live *P. acidilactici* JM01 (at the final concentrations of 1 × 10^6^, 1 × 10^7^, and 1 × 10^8^ CFU/mL) were added. After 3 h incubation, total RNA from RAW 264.7 cells was extracted using TRIzol reagent according to the manufacturer's instructions. Complementary DNA was synthesized, and RT‐PCR was conducted to quantify IL‐1β, IL‐6, TNF‐α, and MCP‐1 genes, normalized to β‐actin (Ahn, Lee, Lee, Kim, and Lee 2023).

### 2.11 Statistical Analysis

All experiments were performed in triplicate and the data expressed as mean ± SD (standard deviation). Statistical significance was assessed using one‐way ANOVA (*p* < 0.05; IBM SPSS ver. 25.0, IBM, Chicago, IL, USA).

## 3 Results and Discussion

### 3.1 Identification of Bacteriocin‐Producing LAB

**FIGURE 1:**
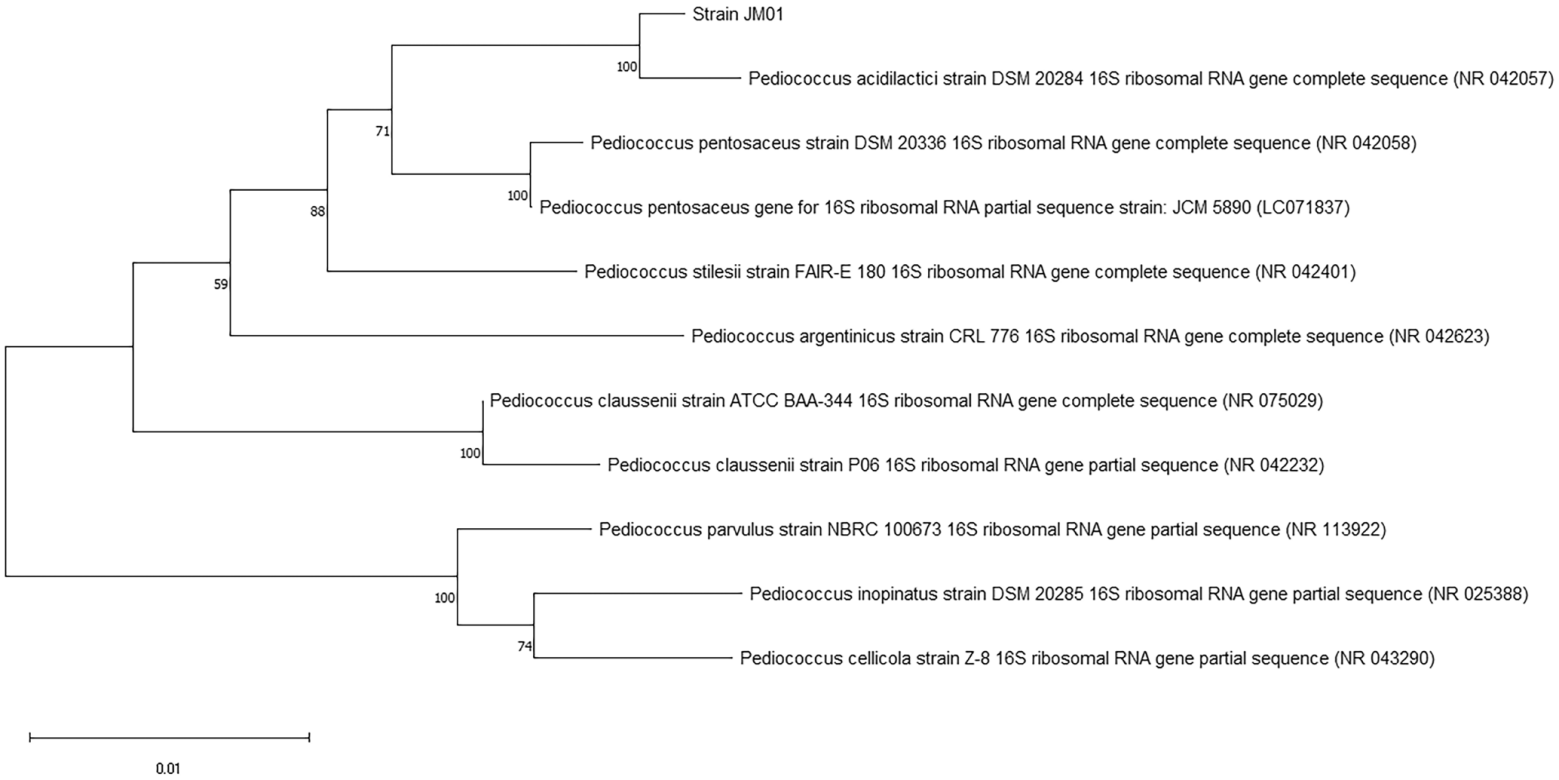
Phylogenetic analysis of sequence from the 16S rRNA gene of the isolate. The scale bar represents 0.01 substitution per nucleotide position.

The isolate exhibiting antimicrobial activity against *Lis. monocytogenes* ATCC 15313 was selected using the ‘Triple‐layer agar method’ from Tarak, a traditional Korean fermented milk. This isolate is a gram‐positive coccus arranged in tetrads and is catalase negative. The API 50 CHL kit results demonstrated 98.8% homology with *P. acidilactici* (data not shown). Additionally, 16S rRNA sequence analysis revealed that the isolate shared 99% homology with the *P. acidilactici* strain DSM 20284 16S ribosomal RNA gene (complete sequence; accession no. NR 042057.1) (Figure 1). Consequently, we designated the isolate as *P. acidilactici* JM01. Previous studies on the microbial community of Tarak identified predominant microorganisms including *Leuconostoc citreum* , *Lactiplantibacillus plantarum*, *Lactococcus lactis* , *Saccharomyces cerevisiae* , among others. *P. acidilactici* was also present in Tarak but was not the dominant species (Lim et al. 2013; Yoon and Shin 2018). An earlier study isolated bacteriocin‐producing *Streptococcus thermophilus* from Tarak; however, in‐depth studies into the characteristics of the bacteriocin were not conducted (Lee et al. 2010). To the best of our knowledge, this study is the first to investigate bacteriocin‐producing lactic acid bacteria and their probiotic traits from Tarak.

### 3.2 Growth Kinetics and Bacteriocin Production

**FIGURE 2:**
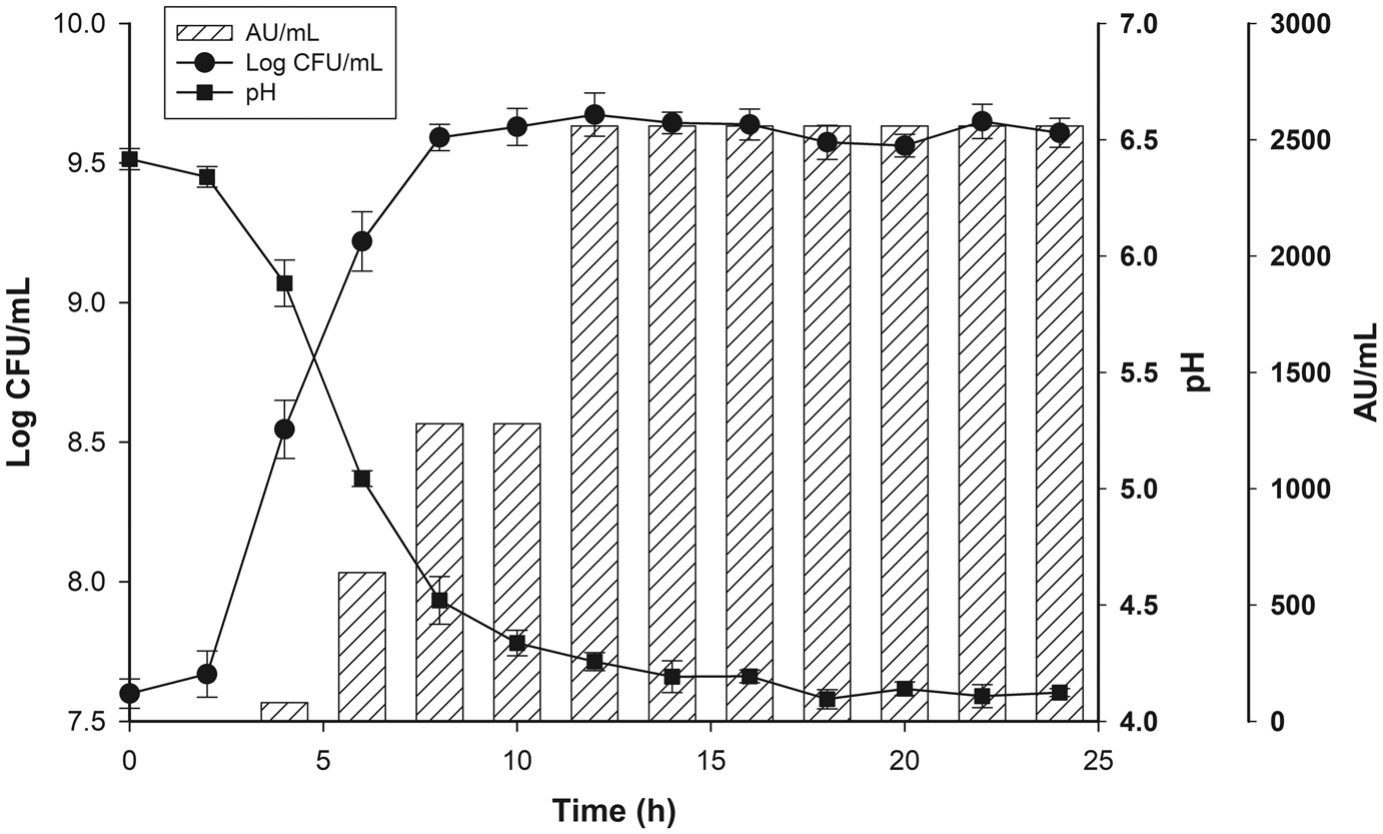
Growth kinetics (‐●‐), bacteriocin production (‐▨‐) of *P. acidilactici* JM01 and the changes in pH of the culture (‐■‐). The error bars represent standard deviation.

Typically classified as primary metabolites, bacteriocins are predominantly produced during the exponential growth phase of bacteria (Figure 2) (Pujato et al. 2024). In our study, *P. acidilactici* JM01 commenced bacteriocin production in the midst of the exponential phase (4 h), maintaining peak activity from 12 to 24 h (2560 AU/mL). Concurrently, pH levels dropped from 6.42 to 4.12 during incubation. Given the general classification of bacteriocin, the compound produced by *P. acidilactici* JM01, as isolated in this research, is likely a primary metabolite. Several studies report similar findings, with bacteriocins from LAB being synthesized during the exponential phase and categorized as primary metabolites (da Silva et al. 2022; Martín et al. 2023; Zangeneh et al. 2020).

### 3.3 Characteristics and Molecular Weight Determination of Bacteriocin JM01

The antimicrobial compound produced by *P. acidilactici* JM01 was confirmed to be proteinaceous by subjecting the CFS to various enzymatic treatments (Table 2). Complete inactivation of antimicrobial activity occurred after exposure to proteolytic enzymes such as trypsin, pronase E, α‐chymotrypsin, and proteinase K, identifying the compound as bacteriocin. Other enzymes did not affect the antimicrobial activity of bacteriocin JM01, indicating that proteins exclusively play a role without carbohydrate or lipid moiety in inhibiting other microorganisms. In contrast, other studies, including our previous work, have isolated amylase‐sensitive bacteriocin‐producing LAB characterized by a glycoprotein nature (Ahn et al. 2017; Ahn, Lee, Lee, and Lee 2023).

**TABLE 2:** Effect of enzymes, temperature, pH, detergents and solvents on the activity of bacteriocin JM01.

Treatment	Bacteriocin activity^a^
Enzyme	
Trypsin	−
Pronase	−
α‐chymotrypsin	−
Proteinase K	−
α‐amylase	++++
Lipase	++++
Catalase	++++
RNase A	++++
pH	
2–9	++++
11	+++
12	−
Heat	
60°C for 10 min	++++
60°C for 30 min	++++
80°C for 10 min	++++
80°C for 30 min	++++
100°C for 10 min	++++
100°C for 30 min	+++
121°C for 15 min	+
Detergent (w/v)	
SDS (1%)	+++
SDS (2%)	+++
Tween 20 (1%)	++++
Tween 80 (2%)	++++
Solvent^b^	
Ethanol	++++
Methanol	++++
Isopropanol	++++
Chloroform	++++
Acetone	++++

^a^ −, absence of inhibition zone; +, 1–5 mm; ++, 6–10 mm; +++, 11–15 mm; ++++, > 16 mm (diameter of inhibition zone).

^b^ Solvents were added with CFS at a 1:1 ratio (w/v).

Bacteriocin JM01 demonstrated remarkable stability of its antimicrobial activity across a wide range of pH values (pH 2–11), indicating its robustness in both acidic and alkaline environments. The sustained structural integrity of bacteriocin JM01 suggests that fluctuations in pH do not significantly compromise its configuration, thereby enhancing its potential for versatile applications under diverse conditions. In addition to its remarkable pH stability, bacteriocin JM01 retained its antimicrobial efficacy even after exposure to autoclaving, a procedure that typically subjects substances to elevated temperatures and pressures. The observed thermal stability of bacteriocin JM01 is likely attributable to specific molecular features, including a high concentration of proline residues known to confer structural stability. Stability may also result from the formation of salt bridges, hydrogen bonds, and interactions with polar surface residues (Ansari et al. 2018; Kamaneewan et al. 2022; Prajapati et al. 2007).

The structural integrity of proteins, including antimicrobial peptides such as bacteriocin JM01, can be significantly influenced by detergents and organic solvents. These agents are known to interact with protein molecules, potentially disrupting their native three‐dimensional structure. Such structural alterations can result in the unfolding or misfolding of the protein, which may, in turn, lead to a reduction or loss of its biological activity. Conversely, specific detergents or solvents might stabilize the protein structure, thereby enhancing its antimicrobial properties (Ansari et al. 2018; Kaya Şen and Kati 2023). Thus, the impact of detergents and solvents on the stability and functionality of antimicrobial proteins is complex and contingent upon the chemical nature of the agent and the particular structural characteristics of the protein in question. Understanding these interactions is crucial for optimizing the use of bacteriocins in various applications. Contrary to our study, in which the treatment of detergents and organic acids had little or no lowering of the antimicrobial activity, several studies have reported an enhancement of activity after treatment with detergents and organic solvents (Ansari et al. 2018; Deraz et al. 2007; Jawan et al. 2021).

**FIGURE 3:**
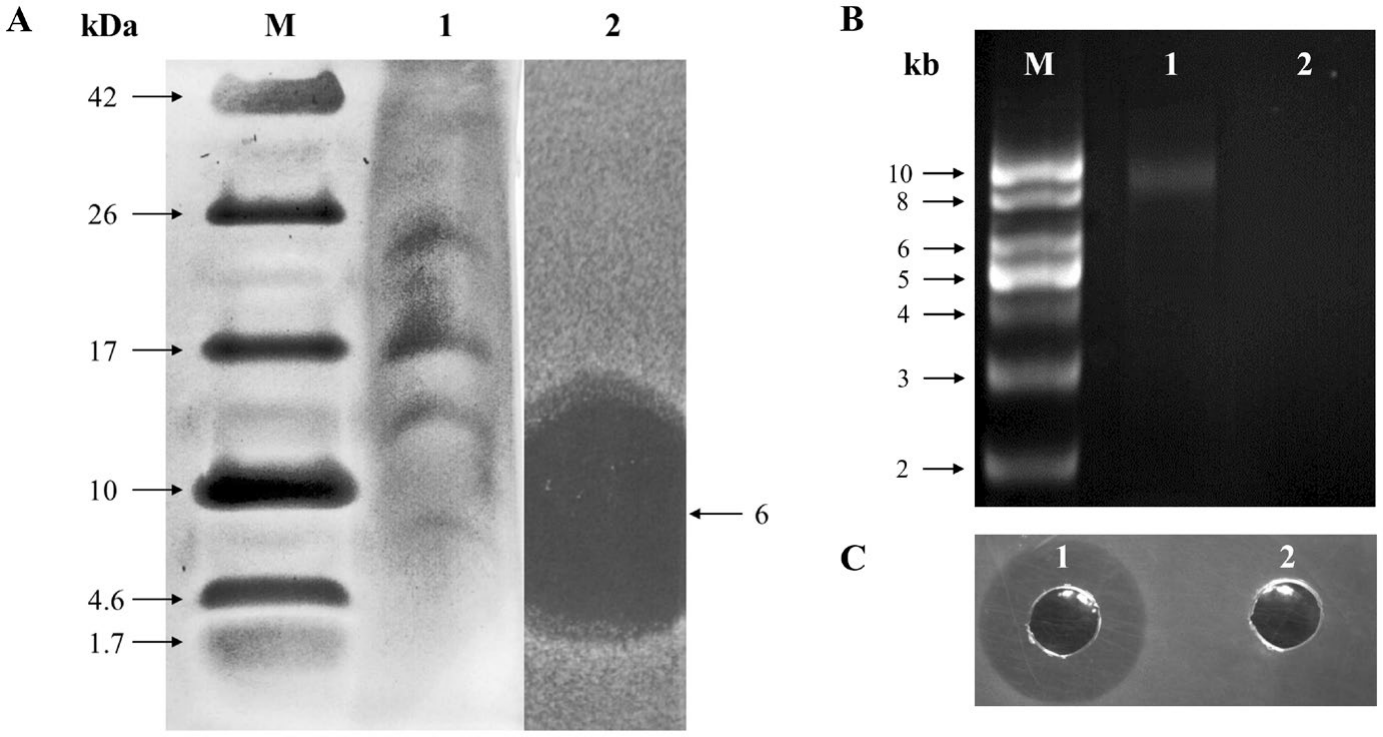
Molecular weight analysis and plasmid‐associated antimicrobial activity of bacteriocin from *P. acidilactici* JM01. (A) Tricine SDS‐PAGE of partially purified bacteriocin; silver‐stained gel. Lane M: Molecular weight marker (Spectra Multicolor Low Range Protein Ladder, Thermo Scientific); lane 1: JM01 bacteriocin; lane 2: Activity overlay with *Listeria monocytogenes* ATCC 19111. (B) Plasmid profiles of JM01 wild type (lane 1) and plasmid‐cured mutant (lane 2); lane M: 1 kb DNA ladder (Elpis Biotech). (C) Antimicrobial activity against *L. monocytogenes* by JM01 wild type (zone 1) and plasmid‐cured mutant (zone 2).

Tricin SDS‐PAGE was conducted to determine the molecular weight of the bacteriocin (Figure 3A), estimated by overlaying the medium pre‐seeded with the indicator strain *Lis*. *monocytogenes* ATCC 15313 onto the SDS‐PAGE gel. Based on the location of the inhibition zone on the gel, the molecular weight of bacteriocin JM01 was identified as 6 kDa, which is larger than the bacteriocins typically produced by *P. acidilactici* . Previous studies have shown that the molecular weights of purified and partially purified bacteriocins typically range from 2.5 to 4 kDa (Dhanda et al. 2023; Villarante et al. 2011; Wang et al. 2015). In this study, the estimation was made using a partially purified sample, indicating that similar protein sizes may co‐precipitate with the bacteriocin during the ammonium persulfate precipitation step. Therefore, further analysis to accurately determine the molecular weight using purified bacteriocin JM01 by reverse phase‐high performance liquid chromatography (RP‐HPLC) is necessary.

### 3.4 Plasmid Study

The location of the gene encoding the bacteriocin was determined by generating a bac mutant through plasmid curing with novobiocin. Figure 3 shows the plasmid profile (Figure 3B) and the results of the agar well diffusion assay for the Bac^+^ (lane 1) and Bac^−^ (lane 2) strains (Figure 3C). The Bac^−^ mutants exhibited a loss of approximately 9 kb in the plasmid, resulting in the loss of the bacteriocinogenic phenotype. Consequently, the plasmid linkage responsible for bacteriocin production in *P. acidilactici* JM01 was identified. Earlier studies confirmed that the bacteriocin‐associated gene in *P. acidilactici* is located within the plasmid, which has a size of 9 kb (Ahn et al. 2017; Marugg et al. 1992; Millette et al. 2008).

### 3.5 Antimicrobial Spectrum

*P. acidilactici* JM01 demonstrated a broad spectrum of antimicrobial activity against a variety of indicators in both deferred and agar well diffusion assays (Table 1). In the deferred assay, the isolate suppressed the growth of gram‐positive bacteria, including *Lis. monocytogenes*, *Pr. acnes*, *S. aureus* , *Str. mutans*, and all tested gram‐negative pathogens, such as *E. coli* O157:H7, *Cr. sakazakii*, *K. pneumoniae* , *Ps. aeruginosa*, *Sh. sonneii*, and *Salmonella* species. However, it did not exhibit any antagonistic effects against yeast strains. Conversely, the antimicrobial effects in the agar well diffusion assay, which represent the action of bacteriocin, were restricted to only gram‐positive bacteria. Among the gram‐positive indicators, bacteriocin JM01 markedly inhibited the growth of *Lis. monocytogenes*, a foodborne pathogen prevalent in dairy and meat products.

Bacteriocin is generally considered to inhibit only gram‐positive bacteria, not gram‐negative cells, as their outer membrane acts as a permeability barrier (García‐Ruiz et al. 2014). Consequently, the inhibition observed in the deferred assay against gram‐negative pathogens might be attributable to the production of other antimicrobial metabolites such as organic acids, hydrogen peroxide, or diacetyl, rather than to bacteriocin (Ahn et al. 2017; Ahn, Lee, Lee, and Lee 2023).

### 3.6 Mode of Action

**FIGURE 4:**
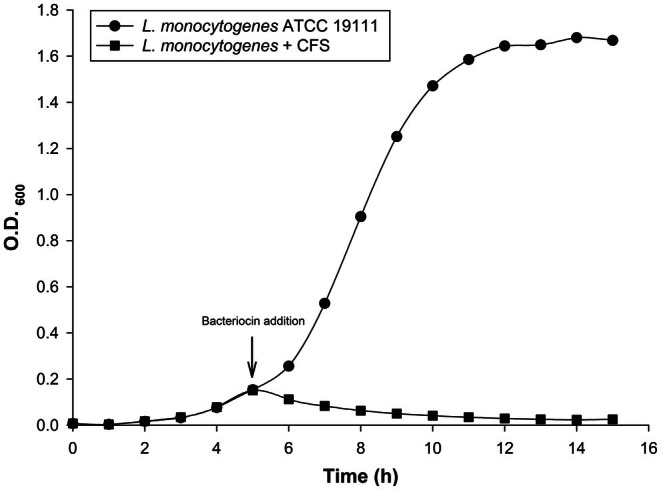
Effect of bacteriocin JM01 on the growth of *L. monocytogenes* ATCC 19111 (‐●‐: control, ‐■‐: Bacteriocin added).

The addition of CFS containing bacteriocin JM01 (2560 AU/mL) to a 5 h‐old (early exponential phase) culture of *Lis*. *monocytogenes* ATCC 15313 resulted in significant growth inhibition (Figure 4). The optical density (OD_600_) decreased from 0.151 (5 h) to 0.025 (15 h), suggesting a. This immediate decrease in optical density indicates that the mode of action of bacteriocin JM01 could be bactericidal, particularly bacteriolytic (cell‐lytic) mode of action for bacteriocin JM01 (Wolden et al. 2023). According to our findings in the antimicrobial spectrum and characteristics of bacteriocin JM01, it was classified as class II. Contrary to expectations, the bacteriocin's mode of action against the indicator strain was similar to that of class IIIa, with a molecular weight > 10 kDa and cell lysis promoted by cell wall hydrolysis (Bisht, Das, and Navani 2024; Hernández‐González et al. 2021). Bacteriocin PA‐1 from *P. acidilactici* PAC 1.0 also demonstrated bacteriolytic activity against *Lis*. *monocytogenes* (Chikindas et al. 1993). Similar findings have been reported (Bhunia et al. 1988; Das et al. 2021). Therefore, further investigations, including observing changes in the cell wall of the indicator strain over time post‐treatment with bacteriocin JM01 via field emission‐scanning electron microscopy and transmission electron microscopy, are warranted.

### 3.7 Probiotic Properties of *P. acidilactici* JM01

The human stomach and small intestine present significant barriers for ingested bacteria due to their low pH and the presence of bile salts, respectively (Wang et al. 2018). Thus, survival during transit through the upper gastrointestinal tract is crucial for probiotic bacteria. The pH of the stomach typically ranges from 1.5 to 6, usually between 2.5 and 3.5 (Huang and Adams 2004), and bile salt concentrations of 0.15%–0.3% are optimal for bile tolerance testing (Frappier et al. 2022; Goldin and Gorbach 1992).

The survival rate of *P. acidilactici* JM01 under acidic and bile conditions is presented in Table 3. *P. acidilactici* JM01 exhibited good tolerance at pH 3.0–4.0 with no significant reduction in viability; however, there was a notable decrease in survival at pH 2.5, recording 71.22% ± 3.97% viability after 3 h of exposure, which was statistically significant (*p* < 0.05). When exposed to varying concentrations of bile, strain JM01 maintained a high survival rate of 90%, even at a bile salt concentration of 2%. Prior studies have shown that *Pediococcus* species and other lactic acid bacteria possess strain‐dependent acid and bile tolerance. *P. pentosaceus* OZF, isolated from human breast milk, experienced a viability reduction of more than 2 log CFU/mL at pH 3.0 and total loss of viability at pH 2.0 after 3 h (Osmanagaoglu et al. 2010). Nonetheless, among all the isolates, *P. acidilactici* SW 05 exhibited the strongest survival at pH 2.0 (Oh and Jung 2015). Given the typical pH and bile salt concentration in the human stomach and small intestine, *P. acidilactici* JM01 showed commendable acid and bile resistance, suggesting its potential to survive passage through the human GI tract.

**TABLE 3:** The survival rate of *P. acidilactici* JM01 to acid, bile conditions, and adhesion ability toward epithelial cells.

Acid	pH 1.5	Viable cell number (Log10 CFU/mL)	0 h	7.67 ± 0.19
			1.5 h	N.D
			3 h	N.D
		Survival rate (%)		0
	pH 2.0	Viable cell number (Log10 CFU/mL)	0 h	8.57 ± 0.12
			1.5 h	N.D
			3 h	N.D
		Survival rate (%)		0
	pH 2.5	Viable cell number (Log10 CFU/mL)	0 h	8.46 ± 0.12
			1.5 h	7.78 ± 0.10
			3 h	6.03 ± 0.33
		Survival rate (%)		71.22 ± 3.97a
	pH 3.0	Viable cell number (Log10 CFU/mL)	0 h	8.56 ± 0.06
			1.5 h	8.59 ± 0.05
			3 h	8.64 ± 0.03
		Survival rate (%)		100.98 ± 1.00b
	pH 3.5	Viable cell number (Log10 CFU/mL)	0 h	8.31 ± 0.09
			1.5 h	8.35 ± 0.04
			3 h	8.37 ± 0.07
		Survival rate (%)		100.70 ± 1.09b
	pH 4.0	Viable cell number (Log10 CFU/mL)	0 h	8.34 ± 0.05
			1.5 h	8.34 ± 0.02
			3 h	8.32 ± 0.04
		Survival rate (%)		99.76 ± 0.62b
Bile salt	0.30%	Viable cell number (Log_10_ CFU/mL)	0 h	8.42 ± 0.11
			2 h	8.34 ± 0.09
			4 h	8.71 ± 0.08
		Survival rate (%)		97.17 ± 1.05^c^
	0.60%	Viable cell number (Log_10_ CFU/mL)	0 h	7.78 ± 0.14
			2 h	7.55 ± 0.17
			4 h	7.41 ± 0.13
		Survival rate (%)		95.23 ± 0.58^b^
	1%	Viable cell number (Log_10_ CFU/mL)	0 h	7.52 ± 0.10
			2 h	6.87 ± 0.12
			4 h	6.80 ± 0.14
		Survival rate (%)		90.32 ± 0.69^a^
	2%	Viable cell number (Log_10_ CFU/mL)	0 h	6.90 ± 0.20
			2 h	6.22 ± 0.28
			4 h	6.24 ± 0.25
		Survival rate (%)		90.41 ± 1.03^a^
Adhesion toward epithelial cell	Caco‐2	Viable cell number (Log_10_ CFU/mL)	0 h	9.62 ± 0.13
			1 h	6.89 ± 0.08
		Adhesion ability (%)		71.58 ± 0.86
	HT‐29	Viable cell number (Log_10_ CFU/mL)	0 h	9.62 ± 0.13
			1 h	6.93 ± 0.08
		Adhesion ability (%)		72.05 ± 0.80

*Note:* Lowercase letters (series “a–c”) indicate significant (Duncan's range test, *p* < 0.05) differences in the column of the same experiment.

Abbreviation: N.D., non‐detected.

The ability of probiotic bacteria to adhere to human intestinal epithelial cell lines, such as Caco‐2 or HT‐29 cells, is considered essential for providing health benefits. The adhesion capacity of the isolate was evaluated using both Caco‐2 and HT‐29 cells, as presented in Table 3. *P. acidilactici* JM01 exhibited enhanced adhesion to HT‐29 cells compared to Caco‐2 cells across all initial counts. The isolate demonstrated 72.05% adhesion to HT‐29 cells at an initial concentration of 10^9^ CFU/mL, representing the highest adhesion rate recorded under the tested conditions. Conversely, an adhesion capacity of 71.58% was observed in Caco‐2 cells at the same initial concentration of 10^9^ CFU/mL. Previous studies have documented the propensity of lactic acid bacteria to adhere more effectively to HT‐29 cells than to Caco‐2 cells (Fonseca et al. 2021; Sharma and Kanwar 2017). In vitro studies demonstrating a high capacity for adhesion to epithelial cells might predict actual colonization in the human colon (Shivani and Sathiavelu 2024). The ability of probiotics to adhere to epithelial cells is attributed to the hydrophobic nature of the cell surface and the presence of extracellular compounds. In this study, we focused solely on an adhesion assay; consequently, additional analyses of cell surface hydrophobicity and auto‐aggregation properties would be necessary.

### 3.8 Anti‐Inflammatory Effect of *P. acidilactici* JM01

**FIGURE 5:**
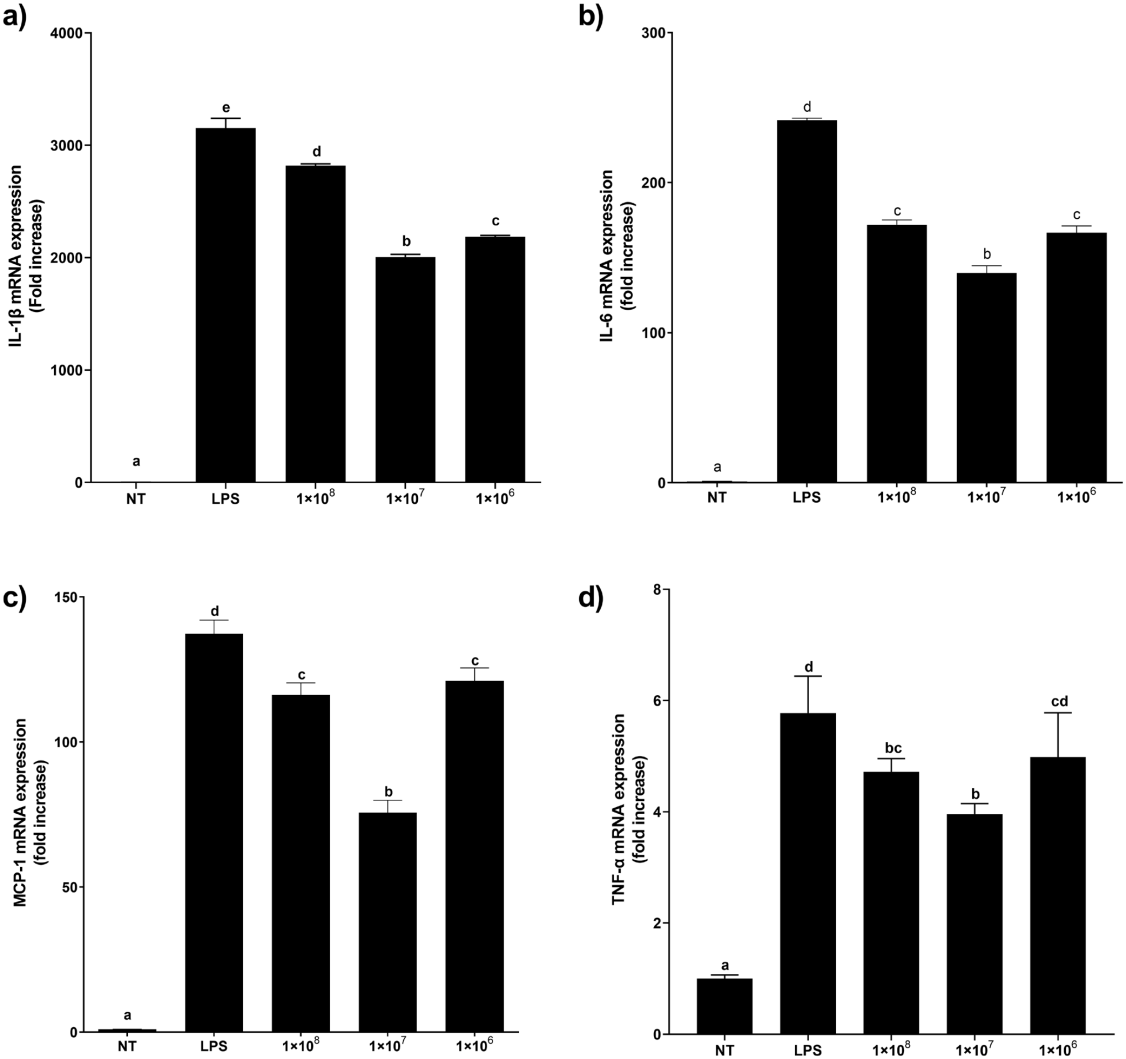
Relative anti‐inflammatory cytokine expression in RAW 264.7 cells on treatment with LPS and *P. acidilactici* JM01 by RT‐PCR. (a) IL‐1β; (b) IL‐6; (c) TNF‐α; (d) MCP‐1.

Probiotics are live bacteria that promote the modulation of gut microbiota balance, benefiting the host and its immune system (Ahn, Lee, Lee, Kim, and Lee 2023; Cristofori et al. 2021; Hao et al. 2024). Lipopolysaccharide (LPS) is a complex macromolecule located in the outer membrane of Gram‐negative bacteria, closely related to immune system activity and exclusively recognized by toll‐like receptor 4 (TLR 4) to induce inflammation; its mechanisms of inflammatory response are well documented (Kim et al. 2024; Mao et al. 2024). In this study, the anti‐inflammatory effect was evaluated using RAW 264.7 cells treated with live *P. acidilactici* JM01 at 10^6^–10^8^ CFU/mL and LPS (1 μg/mL) concurrently (Figure 5). No cytotoxicity to RAW 264.7 cells was observed at the concentrations tested in the MTT assay (data not shown). Treating cells with only LPS significantly increased cytokines, including IL‐1β, IL‐6, TNF‐α, and the chemokine MCP‐1, at the mRNA level. Yet, combining live *P. acidilactici* JM01 with LPS treatment reduced expressions of these cytokines and the chemokine at the mRNA level. Unexpectedly, reductions in cytokine and chemokine mRNA expressions were not observed to be dose dependent. Specifically, the treatment with 10^7^ CFU/mL of *P. acidilactici* JM01 demonstrated the most pronounced anti‐inflammatory effect. With IL‐1β, treatment with 10^7^ CFU/mL resulted in a 36.41% decrease in LPS‐induced IL‐1β expression, while the 10^8^ CFU/mL and 10^6^ CFU/mL treatments resulted in decreases of 10.6% and 30.75%, respectively. Moreover, IL‐6 expression declined by 42.14% with 10^7^ CFU/mL treatment; TNF‐α expression was reduced by 31.49%, and MCP‐1 expression saw a 44.91% reduction. These results suggest that optimal probiotic concentration is critical for maximizing anti‐inflammatory effects. Similar outcomes were observed when treating LPS‐induced RAW 264.7 cells with *P. acidilactici* , which reduced IL‐1β, IL‐6, and TNF‐α expressions through modulation of the NF‐ĸB and JNK/MAPK pathways (Bhatia et al. 2023; Hyung‐Seok et al. 2019). *P. acidilactici* LAB8 treated LPS‐induced mice models exhibited a decrease in cytokine secretion, including IL‐1β, IL‐6, TNF‐α, and IFN‐γ (Bhatia et al. 2022). Concurrently, numerous studies have highlighted the anti‐inflammatory effects of heat‐killed *P. acidilactici* . Specifically, *P. acidilactici* HW01 significantly reduced the levels of IL‐1β, IL‐6, and MCP‐1 in LPS‐induced macrophage cells, demonstrating greater efficacy than *Lb. rhamnosus* GG (Ahn, Lee, Lee, Kim, and Lee 2023). We examined the bacteriocinogenic *P. acidilactici* JM01 for its anti‐inflammatory effects by measuring cytokine and chemokine mRNA expression in an LPS‐induced inflammation model. Further research, including ELISA assays to quantify protein levels of secreted cytokines and Western blot assays to investigate the modulated anti‐inflammatory signaling pathways by this strain, as well as in vivo trials, are essential to further confirm the bioavailability of *P. acidilactici* JM01 for use as a probiotic.

## 4 Conclusions

In this study, *Pediococcus acidilactici* JM01, a bacteriocin‐producing strain isolated from Tarak, exhibited strong antimicrobial activity against *Listeria monocytogenes* and several other Gram‐positive pathogens, along with probiotic characteristics such as acid and bile salt tolerance, adhesion to intestinal epithelial cells, and anti‐inflammatory effects. These findings indicate that JM01 has potential as both a natural antimicrobial and a functional probiotic that may contribute to food safety and gut health. Further studies are needed to assess its practical applications as a functional probiotic material, to conduct structural characterization of bacteriocin, and to evaluate its utilization as a bio‐preservative in food processing.

## Author Contributions

**Hyunwoo Ahn:** conceptualization (equal), data curation (lead), formal analysis (lead), methodology (lead), visualization (lead), writing – original draft (lead), writing – review and editing (equal). **Hyun Jun Lee:** conceptualization (equal), data curation (supporting), project administration (lead), resources (lead), supervision (lead), writing – original draft (supporting), writing – review and editing (equal).

## Conflicts of Interest

The authors declare no conflicts of interest.

## Data Availability

The data that support the findings of this study are available from the corresponding author upon reasonable request.
